# A Brief Discussion of the Economic Importance of the Most Common Adult Cestodes of Man in the United States (Taenia Saginata, Dibothriocephalus Latus, Hymenolepis Nana, and Taenia Solium); with Report of Two Cases

**Published:** 1910-02

**Authors:** Charles A. Pfender

**Affiliations:** Washington, D. C.


### TEXAS MEDICAL JOURNAL.

Established July, 1885

F. E. DANIEL, M. D., - - - - - - Editor, Publisher and Proprietor

Published Monthly.—Subscription $1.00 a Year.

VOL. XXV AUSTIN, FEBRUARY, 1910. No. 8

The publisher is not responsible for the views of the contributors.

### Original Articles.

For Texas Medical Journal.

### A Brief Discussion of the Economic Importance of the Most Common Adult Cestodes of Man in the United States (Taenia saginata, Dibothriocephalus latus, Hymenolepis nana, and Taenia solium); With Report of Two Cases.

BY CHARLES A. PFENDER, M. D., WASHINGTON, D. C.

In the light of recent developments in the study of other animal parasites of man occurring in the United States, tapeworm infection has not received the consideration it merits. The economic importance of the varied symptomatology encompassing general distress, and mental and physical impairment, so frequently associated in this form of helminthiasis, has not been properly appraised.

The prevailing idea that the Federal meat inspection system of the present day suffices to prevent all danger of infection is not correct. Under the law, only those establishments which prepare meat food products that are to be shipped interstate are required to submit to Federal inspection. In the larger cities where it is practical to maintain a complete inspection service, supplementing the Federal service if necessary, it is possible to prevent the sale of meat which has not been subjected to a rigid examination by experts and considered suitable for food. There are hundreds of smaller towns and communities in the United

States, however, where this is not the case. In these localities cattle, hogs, lambs, etc., are slaughtered, and the meat sold and consumed without ever a thought of subjecting it to inspection, or a suspicion of possible infection.

The use of raw beef as food is a far more common occurrence in the United States than is usually admitted to be the case. This habit is especially prevalent among the Germans and Poles, although by no means confined to these classes of our population.

We find it is the city practitioner who usually reports a case of tapeworm infection. There are, however, few country practitioners who have not encountered one or more cases of infection with the beef tapeworm, and have successfully caused its expulsion, yet did not deem it worth while to report or publish their observations. I have personal cognizance of a number of just such instances, although my experience has been restricted to certain counties of Texas and to the District of Columbia.

A study of the records of the specimen catalogs of the Zoological Laboratories of the Bureau of Animal Industry of the Department of Agriculture and of the United States Public Health and Marine Hospital Service shows that during the past ten years there have been received for identification and preservation thirty-eight specimens of *Tænia saginata* collected in different parts of the United States. During this same period of time thirty-eight further cases were deemed of sufficient importance to the authors to merit publication in the medical press, and the majority of these were reported during the past five years. A systematic inquiry among the physicians of the United States would, no doubt, reveal a vast number of cases thus far unreported, and would enable us to formulate somewhat accurate deductions as to geographical distribution and prevalence in the different nationalities represented in our country.

The order of the importance of adult tapeworm infection is, perhaps, commensurate with the comparative frequency of occurrence in the United States. By far the heaviest infection is due to *Tænia saginata* (Goeze, 1782), also called the beef or fat tapeworm. This tapeworm, in its adult stage, lives exclusively in the intestinal tract of man. The cystic stage of this parasite resides in the ox and is transmitted to man through eating raw or insufficiently cooked meat containing these cysts. Such a cyst, termed *Cysticercus bovis,* rapidly develops into the adult stage after it has gained admission to the host, and may attain a length of four to twenty feet within a few months. Probably the most remarkable feature in this connection is that the presence of such an animal may not occasion any clinical symptoms whatever. On the other hand, it may give rise to a multitude of morbose manifestations of variable intensity in different individuals. Its most baneful effect is evinced by a lowering of the vitality of the host, and, in some cases, it incurs almost total temporary physical disability. As regards life, however, it is by no means a menace. Fatal cases have not been reported, and it is likely that the toxic material produced by this parasite, if there be any, is not so destructive as that of the much less common tapeworm next in the order of economic importance.

The *Dibothriocephalus latus* (Linnæus, 1748), or broad tapeworm, also commonly called Russian or Irish tapeworm for geographical reasons, is rapidly being recognized as an important factor in the United States. The first case of this kind was reported in 1879, by J. T. Walker, of Philadelphia. Since that time forty-two cases have been brought to the attention of the medical profession. It was demonstrated in all of these that the infection had been brought to this country by immigrants, with one exception. This case of *Dibothriocephalus latus* infection occurred in a child, aged 4 years, born in Ely, Minnesota. It was shown conclusively that this child had never been out of the United States, nor had it eaten of imported fish. This is, therefore, the *first undoubtedly autochthonous case* of *Dibothriocephalus latus* infection reported in the United States. Nickerson, who reported this case, is authority for the statement that he found plerocercoids of this tapeworm in American fish caught in the Great Lakes. This would explain the source of infection. The reason infection is not more common in our country lies, no doubt, in the improved methods of cooking fish.

The cysticercus stage, or plerocercoid, lives only in fish, such as the pike, carp, salmon and trout. As it does not live in warm-blooded animals, there is no danger of autoinfection. In certain localities of Europe parts of these fish are eaten raw, and infection is thus favored. Infection may take place through the ingestion of raw, salted, or smoked uncooked fresh-water fish which harbor these plerocercoids.

The broad tapeworm is a rather common parasite of man in some parts of Europe. Certain classes of the population are largely dependent on a fish diet in the regions where this tapeworm abounds, and, as we have many immigrants from these localities, we can account for the introduction of this species into the United States.

The disturbances which this tapeworm produces in man, like those of *Tænia saginata* infection, are usually not very marked. Some impairment of the general health is always incurred, however, while in some instances gastric and nervous manifestations of great severity are noted. The most important pathological factor is a pronounced type of pernicious anemia which occurs in a small percentage of cases. This is probably due to the absorption, by the human organism, of a toxic substance which either exists latent in the worm, or is manufactured by it under certain conditions. When we consider, however, that only a small number of cases infected with this tapeworm develop pernicious anemia, it is likely, as has been suggested by Schaumann, that a hereditary tendency to anemia exists in these cases. In a case reported by W. Gilman Thompson, in 1905, the hemoglobin had been reduced to 20 per cent, red cells 608,000, leukocytes, 2000. Seven months after the expulsion of the parasites the blood count showed hemoglobin 98 per cent, red cells 5,980,000, leukocytes 10,000, and the patient had completely recovered. Foreign writers have reported a number of fatal cases, but so far none has been observed in the United States.

Only one specimen of this tapeworm is usually present, but multiple infection is not uncommon. Willson found at least two worms, neither of which measured less than 35 feet in length. R. L. Wilson found thirteen heads, and the entire mass totaled 319 feet. The growth of the worm is quite rapid. It has been found capable of producing from fifteen to thirty segments a day, and has been reported as existing in the intestinal tract of man as long as thirty-five years. In Willson's case it had been present at least fifteen years.

The following table contains forty-two cases which I have collected from the literature.* It will be observed from the wide range of infection that the geographical distribution and the comparative frequency in certain localities is a more or less accurate index to the residence of the immigrants from those parts of Europe where fish is the main article of food:

---

*Shivers, in 1900, reported a case of tapeworm infection which he designated as being "of the *Bothriocephalus latus* variety." I rather incline to the belief that he was dealing with *Tænia saginata*.

| Case | Nativity | Reported | Locality | Author |
|---|---|---|---|---|
| 1 | Sweden | 1879 | Pennsylvania | J. T. Walker. |
| 2 | Finland | 1891 | Pennsylvania | F. A. Packard. |
| 3 | Finland | 1896 | New York | J. Haglestam. |
| 4 | Finland | 1896 | Michigan | J. Haglestam. |
| 5 | Europe | 1898 | Colorado | G. Dock. |
| 6 | Europe | 1898 | Texas | G. Dock. |
| 7 | Europe | 1898 | Texas | G. Dock. |
| 8 | Russia | 1902 | Pennsylvania | R. N. Willson. |
| 9 | Russia | 1902 | Pennsylvania | J. McFarland. |
| 10 | Russia | 1902 | Pennsylvania | D. Riesman. |
| 11 | Scandinavia | 1902 | Pennsylvania | Stengel. |
| 12 | Europe | 1902 | Pennsylvania | Stengel. |
| 13 | Finland | 1903 | New York | Janeway. |
| 14 | Sweden | 1903 | New York | Ewing. |
| 15 | Russia | 1903 | New York | W. N. Berkeley. |
| 16 | Russia | 1903 | New York | W. N. Berkeley. |
| 17 | Russia | 1903 | New York | W. N. Berkeley. |
| 18 | Russia | 1903 | New York | W. N. Berkeley. |
| 19 | Pennsylvania (Traveled in Europe) | 1903 | Pennsylvania | H. M. Bellows. |
| 20 | Finland | 1904 | Pennsylvania | *W. L. M. Coplin. |
| 21 | Sweden | 1904 | Colorado | *J. M. Walker. |
| 22 | Finland | 1904 | Pennsylvania | Edsall. |
| 23 | Finland | 1904 | Pennsylvania | Edsall. |
| 24 | Russia | 1904 | Massachusetts | Edsall (Tiletston). |
| 25 | Russia | 1904 | Massachusetts | Edsall (Tiletston). |
| 26 | Russia | 1905 | Wash., D. C | *Hyg. Laboratory |
| 27 | Finland | 1905 | New York | W. G. Thompson. |
| 28 | United States (Finnish parents) | 1906 | Minnesota | W. S. Nickerson. |
| 29 | Finland | 1906 | Minnesota | W. S. Nickerson. |
| 30 | Finland | 1906 | Minnesota | W. S. Nickerson. |
| 31 | Japan | 1906 | Montana | W. S. Nickerson. |
| 32 | Town of Baltic Sea | 1907 | Ohio | Van Nuys. |
| 33 | Sweden | 1907 | New York | H. L. Lynah. |
| 34–40 | Poland | 1908 | New York | F. Huber. |
| 41 | Russia | 1908 | New York | C. O. Boswell. |
| 42 | Germany | 1908 | Louisiana | R. L. Wilson. |

*Taken from catalogue of Zoological Laboratory of the Public Health and Marine Hospital Service, Washington, D. C.

Forty of the forty-two cases were foreigners. Case 19 had traveled in Europe and had probably gotten infected while abroad. The only indigenous case was in a child of Finnish parents. We find from the accounts given that 11 were Russians, 10 were Finlanders, 7 were Poles, 4 were Swedes, 1 a Scandinavian, 1 a Japanese, 1 a German, the others not being designated further than as Europeans.

We note that seventeen cases were observed in New York, eleven in Pennsylvania, three in Minnesota, two each in Texas, Massachusetts and Colorado, and one each in Louisiana, Michigan, Montana, Ohio, and Washington, D. C.

The two species of tapeworms discussed above are known to attain large proportions, varying in length from a few feet to many yards. It is, therefore, exceedingly interesting to note that the cestode next in order in point of frequency and importance is of miniature proportions. In fact, Dr. Stiles is convinced that it is the most common tapeworm throughout the South. This is the dwarf tapeworm or *Hymenolepis nana* (Siebold, 1852). It attains a length of 5 to 45 millimeters, and a width of 0.5 to 0.9 of a millimeter. It is composed of 100 to 200 minute segments, and is situated, in multiple numbers, in the upper portion of the ileum.

The presence of this tapeworm had frequently been reported in other countries, but it was brought to the attention of the medical profession of the United States, for the first time, in 1902, by Dr. John T. Moore, of Galveston, Texas. A case had been reported by Dr. Spooner, of Philadelphia, as early as 1873, but no special significance was attached to it at that time, nor was its more common occurrence suspected.

In spite of the fact that this tapeworm is of diminutive size it produces considerable disturbance in man, particularly as it occurs most frequently in children, and usually in large numbers, hundreds and even thousands of worms being present in a single patient. The manner of infection still remains unsolved. It is not unlikely, however, that it occurs through the ingestion or handling of food soiled by excrements of mice and rats.

A total of thirty cases have been reported in the literature of the United States to date. With the exception of Spooner's case, they were all reported during the past six years, as a glance at the following table will show:

| Date | Author | No. of cases | Locality |
|---|---|---|---|
| 1873 | Spooner | 1 | Philadelphia, Pa. |
| 1903 | J. T. Moore | 1 | Galveston, Texas. |
| 1903 | C. W. Stiles | 1 | Charleston, S. C. |
| 1903 | C. W. Stiles | 3 | Macon, Ga. |
| 1903 | L. E. Magnenat | 4 | Amarillo, Texas. |
| 1903 | Hyg. Laboratory | 12 | Washington, D. C. |
| 1904 | H. M. Hallock | 2 | Fort Porter, N. Y. |
| 1906 | W. H. Deaderick | 4 | Marianna, Ark. |
| 1906 | S. W. Lambert | 1 | New York City. |
| 1907 | W. H. Deaderick | 1 | Arkansas. |
| | *Total | 30 | |

*In addition to the above thirty cases, "several cases" are credited to Dr. De Velling, of Jackson, Mississippi. A case by Dr. H. F. Long, of

It seems that the prophecy by Dr. C. W. Stiles, in 1902, to the effect that *Hymenolepis nana* would probably prove to be a much more common and more important factor in the United States than hitherto suspected, is approaching verification. It enjoys a wide geographical distribution; its present known range extends from New York to Texas, and as far westward as Mississippi and Arkansas.

As a result of the study of adult tapeworm infection of man it is difficult to escape the conclusion that *Tænia saginata, Dibothriocephalus latus* and *Hymenolepis nana,* in the order named, comprise the cestodes of greatest economic importance in the United States.

It was held at one time that the pork-measle tapeworm (*Tænia solium* (Linnæus, 1758), which is derived from eating undercooked, under-pickled or under-cured pork infected with the bladder worm or *Cysticercus cellulosæ,* was quite prevalent in this country, but when we consider that during the past ten years only two supposed cases were reported in the literature, none since 1899, we are perhaps justified in relegating this parasite to a place of lesser importance. The term "supposed" is chosen advisedly, for there exist grave doubts as to the correctness of the determination. The case reported in 1899, by Dr. J. L. Hensley, of Marion, Ohio, was probably *Tænia saginata.* The meager description given tallies more with the characteristics of the latter than with those of *Tænia solium.* Further, the author states that "*Tænia solium* is most common in this country," whereas we know that *Tænia saginata* unreservedly pre-empts the position here ascribed to *Tænia solium.* The other case, reported in the same year, by Dr. B. Lapowski, of New York, was probably a true *Tænia solium.* We have no assurance, however, that the determination of the parasite was a correct one. The patient showed no subjective or objective signs of the presence of tapeworm infection and came to treatment for spermatorrhea and prostatorrhea, both of which disappeared upon expulsion of the parasite.

About fifteen years ago Stiles stated that "of all the 300 or more specimens of human tapeworms from patients in various parts of this country, which have passed through my hands during the past three years, three specimens have been *Bothriocephalus latus,* and all the rest have been *Tænia saginata.*" He

Statesville, N. C., is noted in the catalog of the Hygienic Laboratory, for the year 1905. Dr. Stiles further informed me that he know of twenty more unrecorded cases of *Hymenolepis nana* infection which were brought to his notice in the past few years.

adds, further, that "the only adult *Tænia solium* contained in the government and private collections under my charge, is one imported in alcohol from Leipzig"; and, in 1905, he states that during a period of fifteen years (1891-1905) he has not received a single indigenous authentic specimen of *Tænia solium.* Only quite recently did he have occasion to observe this first case of pork tapeworm infection in the United States.

The above statements support our conclusions that the presence of this cestode in man is indeed a rare occurrence in the United States. That it has occurred cannot be denied, as authentic cases are on record, and the cystic stage of this parasite has been found in the hog. The *Cysticercus cellulosæ* of *Tænia solium* is alleged to be present in hogs in some parts of Texas. A number of specimens have also been found by the meat inspection service of the Bureau of Animal Industry. Ransom reports having observed the larva of *Tænia solium* in sheep also. The chief danger in *Tænia solium* infection lies in the possibility of autoinfection, thus producing cysticercosis in man, which has proven that.

In the following table are recorded the cases found in the literature and in the catalogs of the Zoological Laboratories at Washington, D. C., during the past ten years:

| Species of Tapeworm. | Number (1899-1909). |
|---|---|
| *Tænia saginata* | 76 |
| *Dibothriocephalus latus* | 38 |
| *Hymenolepis nana* | 30 |
| *Tænia solium* | 2 ? (Third case unreported). |

There are authentic reports of instances of infection with other varieties of adult cestodes, such as *Hymenolepis diminuta* (Rudolphi, 1819), *Dipylidium caninum* (Linnæus, 1758), and *Tænia confusa* (Ward, 1896), but they occur so rarely that they do not merit further consideration at this time.

*General Symptomatology.*—The symptoms produced by tapeworm infection are based on certain definite causative factors. It would be difficult to decide upon the order of their relative importance. Helminthologists usually classify them as follows:

1. Mechanical obstruction.
2. Local irritation or injury to the intestinal wall, and its influence upon the nervous system.
3. The assimilation of food by the worm which would otherwise go to the host.
4. The absorption of toxins produced by the parasite.

Mechanical obstruction may be a considerable factor in infection with *Tænia saginata, Dibothriocephalus latus* and *Tænia solium* on account of the enormous size which these tapeworms sometimes attain. In the case of infection with *Hymenolepis nana* this factor is brought into play only in those cases where the worms are present in overwhelming numbers.

Irritation and injury to the intestinal wall occurs in all of these varieties of infection. The extent thereof is probably measured by the size and number of the tapeworms present, and by the armature of the different species.

The amount of assimilation of nutrition by tapeworms is problematical. This undoubtedly also depends upon the size and number of worms present.

The elimination of toxic substances appears to play an important role in infection with *Dibothriocephalus latus*, less so in *Tænia saginata* and *Hymenolepis nana*, and is, as far as we know, a negligible factor in *Tænia solium* infection. The severe anemia sometimes encountered when infection with *Dibothriocephalus latus* and *Tænia saginata* obtains may be largely due to the absorption of toxins eliminated by these parasites.* When we consider the lilliputian proportions of *Hymenolepis nana* we are inclined to attribute the serious train of symptoms which sometimes follows in the wake of infection with this parasite to other than mechanical reasons, and, naturally, are led to assume that a toxic material is eliminated by this parasite. This point, however, requires further investigation, as little is known at present in regard to this function.

In a large number of cases no appreciable symptoms are present. In cases of greater intensity there exists no characteristic train of clinical manifestations. The disturbances occasioned by the parasite may range from slight general indisposition to severe gastric and nervous derangement, and may give rise to a multitude of different symptoms in one patient, or show an accentuation of some selective feature in another. In point of medical importance, as evinced by the severity of the individual clinical picture, infection with *Dibothriocephalus latus* merits further consideration. It is not the purpose of this paper to present an array of the symptoms encountered, as these may be readily gleaned from other sources, but we would direct special attention to the fact that the anemia produced in some instances is of a pernicious type, and has claimed several victims. No deaths have been observed in this country thus far, but in the foreign literature one

---

*It is interesting to note that experiments recently made by Fetterdolf, at Philadelphia, with live segments and glycerin extract of *Tænia saginata*, led the author to conclude that this tapeworm secretes an antienzyme, the presence of which greatly retards digestion of fibrin.

encounters reports of several fatalities due to pernicious anemia produced by infection with this tapeworm. In every case reported in the United States the symptoms disappeared entirely after the worm had been expelled.

So far as it was possible to ascertain, no deaths have been reported due to infection with *Tænia solium* or *Tænia saginata.* In a large number of cases of *Tænia saginata* infection, observed in this country, all manner of disturbances and irregularities were noted, but these disappeared promptly after expulsion of the tapeworm.

From a study of the cases of *Hymenolepis nana,* observed in the United States, in which symptoms were noted, Deaderick found that the principal symptoms in order of frequency were as follows:

1. Abdominal pain.
2. Diarrhea.
3. Headache.
4. Nausea.
5. Vomiting.
6. Disturbed appetite.
7. Nervous symptoms.
8. Dysenteric symptoms.
9. Fever.
10. Dyspeptic symptoms.

In some cases symptoms were absent. In others the severity of the clinical picture was directly proportional to the number of worms present, but this was not true in every case; quite heavy infection in a few cases being accompanied by comparatively slight disturbance. Expulsion of the worms was invariably followed by improvement of the general condition of the patient.

*Diagnosis.*—The finding of the ova or of segments of the tapeworms in the feces will establish the diagnosis in every case.

*Prognosis.*—The outlook for life is always good, with the exception of the cases of *Dibothriocephalus* infection associated with the severe anemia. The duration and extent of the anemia will modify the prognosis in these cases. Recovery usually follows expulsion of the worms, and the symptoms disappear. *Tænia saginata,* although without hooks, appears to be the most difficult to expel, and repeated doses of anthelmintics may be necessary to effect entire removal. *Tænia solium* is more readily expelled, and *Dibothriocephalus latus* has not proved very resistant, while *Hymenolepis nana* yields quite readily to the administration of male fern.

*Treatment.*—The treatment is active and prophylactic. The former may be divided into three separate stages:

1. Preparatory.
2. The administration of an anthelmintic.
3. The period of expulsion.

The preparatory treatment consists of proper diet and the administration of a laxative. This is of vital importance if our efforts to expel the parasites are to be crowned with success. We have an illustration of this in case 1, where its omission, no doubt, accounted for the failure to expel the worm.

Male fern is the most common drug used in this country for the expulsion of the large tapeworms, and it has also proved a most successful remedy in the treatment of the dwarf tapeworm. Some purgative other than castor oil should be employed along with it, as castor oil favors the absorption of male fern and exposes the patient to male fern poisoning. Care should be exercised when administering male fern to children. Cusso, kamala, pelletierine tannate, pumpkin seeds, the bark of fresh pomegranate root, etc., have all been employed with varying results in the endeavor to cause expulsion of the large tapeworms.

During the period of expulsion an enema should be administered and a large vessel containing warm water should always be held in readiness for the reception of the worm. The head should always be sought for. It has been recommended that the protruding end of large tapeworms be injected with morphine so as to encourage the animal to relinquish its hold.

The prophylactic measures at our disposal are clearly indicated in those varieties of tapeworms in which the manner of infection is known. In cities, infection with *Tænia saginata* may be prevented by efficient meat inspection service. Cold storage for twenty-one days will effectively destroy the cystericerci in beef. In smaller towns and country districts where a system of meat inspection is not maintained, abstinence from raw or under-cooked beef is a safe method of prevention. The thorough cooking of fish from lakes known to be infected will prevent infection with the *Dibothriocephalus latus*. Infection with *Tænia solium* is prevented by eating none but thoroughly cooked pork. In view of the fact that the intermediate stage has been found in sheep, it may be well to observe this precaution in the case of mutton also, though the parasite is admittedly rare in sheep. It is probable that by avoiding contact with excrements of rats and mice infection with *Hymenolepis nana* will not occur, although Stiles believes that the tapeworm obtained from rats and mice is of a different species.

The two cases of tapeworm infection appended below are of interest on account of the dissimilarity between them in so many features. In one the symptoms were many and severe, while in the other symptoms were absent, beautifully illustrating the somewhat paradoxical statement made by an American authority that the most characteristic feature in tapeworm infection is the lack of characteristic features.

CASE 1.—Male, aged 14 years, complained of having suffered from violent headaches for nearly a year; recurrences daily; frequent epistaxis; ravenous appetite and always hungry; gnawing pains in the abdomen and region of stomach after eating. Pain is especially severe after eating raw cocoanut. Frequent attacks of vertigo and nausea; vomiting occurred upon the slightest provocation; could vomit at will, so long as he "had something in his stomach." Frequent diarrhea.

He was fairly well developed for his age, but pale and anemic; dark circles underneath the eye. Physical examination was negative, with the exception of a faint hemic murmur. Blood and feces were not examined. He was given a hematinic. No improvement during the next two months. He reported that he had found blood in his stools. No hemorrhoids. Examination of the feces showed them to be of a fairly normal consistency, dark in color, streaked with small lines of bright red blood, and containing small white segments. These segments were about half an inch to an inch and a half in length, about three-eighths of an inch wide, and quite motile. They were recognized as tapeworm segments. Subsequent stools examined revealed from three to twenty segments in each stool, and in forty fecal examinations the segments were absent in none. Microscopic examination of one of the segments proved it to be *Tœnia saginata.* Inquiry elicited the fact that raw Hamburg steak was a frequent item of food at the home of the patient. As I was now of the opinion that the infection with this tapeworm accounted for the general constitutional derangement and for the anemia, I made arrangements for the expulsion of the parasite.

The patient was advised not to partake of food the evening before the day of treatment. A glass of milk only was allowed him, and at bed time a laxative administered. A glass of milk was given the next morning and an hour later an ounce of castor oil and coffee was administered. This was followed in ten minutes by half an ounce of fluid extract of male fern, and twenty minutes later another dose of castor oil was given. He was then put to bed. An hour later a saline enema was administered and shortly afterwards the worm, inclusive of the head and neck, was expelled. Shortly afterwards the patient vomited a considerable quantity of dark-green fluid having the characteristic odor of male fern. With the exception of a feeling of great weakness, he suffered no ill-effects from the treatment. He got up during the afternoon and walked down town. His condition began to improve at once. He soon assumed a better color, and within two months he had gained six pounds in weight. *All of the distressing symptoms from which he suffered so much before the expulsion of the worm had disappeared.* The tapeworm was measured on a wet glass plate and found to be about thirteen yards in length. The patient was charged to abstain from eating raw beef, and then dismissed.

He returned about eighteen months later and surprised me with the statement that he had another tapeworm. His distress was not so great as in the first instance, and he informed me that he had been in the habit of looking for segments for some time. As soon as they appeared he came for treatment. He insisted upon taking the male fern at once without any preliminary fast. This was at 2 o'clock in the afternoon. I was not so fully impressed with the importance of observing the preparatory measures then as I am now, and yielded to his importunities. I gave the same dose as before, but no segments came away. He showed no immediate ill-effects from the dosage and left for home. A week later he returned and told me that he had suffered for the entire week from effects of the treatment. The most pronounced symptoms being nausea, anorexia, and great general weakness. No doubt the amount of male fern absorbed produced these toxic symptoms. A month later the usual treatment was carried out and the tapeworm duly expelled. It measured about eighteen feet. No bad effects followed the treatment, and as the patient declared he would forever forego the doubtful pleasure of eating raw beef it is but logical to assume that he had not suffered from another infection, provided he adhered to his assertion.

Case 2.—Mr. N., aged 29, came to me September 2, 1909, with the statement that he believed he had a tapeworm. He admitted having eaten very rare beef in Cuba about eight months ago. An examination of a specimen of feces showed segments and ova of *Tænia saginata.* The patient was somewhat pale, probably due to confinement, his occupation being that of clerk, and he complained of a ravenous appetite. The only real distressing symptom was the mental worry occasioned by the knowledge of the presence of the parasite.

Being somewhat intimidated by a number of reports anent the toxic effects of male fern, some cases having proven fatal, I decided to abstain from the regular doses recommended and employed by foreign writers. I prescribed four capsules of oleoresin of male fern of 15 grains each, one to be taken every fifteen minutes. The usual preparatory treatment was followed. Castor oil was not given, but Cascara evacuant used instead, and given two hours after the administration of male fern. Repeated enemas were employed. No tapeworm was discharged. A week later 20 grains of Tanret's pelletierine tannate was given, preceded and followed by a mild purgative. Still no tapeworm. A week later I advised the patient to get two ounces of pumpkin seeds, macerate them and mix with sugar and drink the draught. In his anxiety to make sure of the removal of the parasite he procured three ounces of the seeds and took most of them. He would have taken all had his system not revolted against taking more than about two and a half ounces. About eight hours later the worm was expelled. He neglected to use the prescribed vessel filled with warm water and almost lost the parasite by way of the sewer. As it was, he collected about twelve yards of tapeworm, but the head was not found. He was quite weak on the following day and developed such an intense headache that he was compelled to lie down. The headache disappeared in a day or two, but his general condition showed very little change. On November 18th, two months later, he returned with the statement that he was again passing segments. This time he was given two drachms of oleoresin of male fern at one dose mixed with simple syrup, followed two hours later by a large dose of Epsom salts. In a few hours he passed several pieces of tapeworm, the longest measuring seven yards. What appeared to be the neck was found, but the head again remained undiscovered, and it is possible that it was not expelled. In this case the symptoms were so slight and vague that had it not been for the occasional discharge of segments observed by the patient it is not likely that the presence of a tapeworm would ever have been suspected.

### BIBLIOGRAPHY.

Adams, S. S., 1902.—Carbolic acid poisoning in a child, resulting from the rectal injection of a solution of the drug for the removal of worms. Tr. M. Soc., Dist. Columb., v. 6, pp. 94-96.

Bellows, Horace M., 1903.—*Bothriocephalus latus.* [Letter dated August 29.] Med. News, Phila., v. 83, Sept. 12, pp. 517-518.

Berkley, William N., 1903.—Case of *Bothriocephalus latus* in a woman immigrant, with remarks on the occurrence of this parasite in America. Med. News, N. Y., v. 83, p. 203.

Boswell, Charles O., 1908.—A case of *Dibothriocephalus latus* infection. Buffalo M. J., v. 63, Apr., pp. 511-516.

Brown, D. J. Milton, 1901.—A case of *Tænia saginata* in a child of twenty-four months, from the use of raw scraped beef for the relief of chronic intestinal disturbance. Pediatrics, v. 12, pp. 90-92.

Davis, G. G., 1900.—Removal of an acutely inflamed appendix containing a segment of a tapeworm. Proc. Path. Soc., Phila., n. s., v. 3, pp. 129-130.

Deaderick, William H., 1906.—*Hymenolepis nana* and *Hymenolepis diminuta*, with report of cases. J. Am. M. Ass., Chicago, v. 47, Dec. 22, p. 2087.

1907.—Notes on the occurrence of *Hymenolepis nana* in the United States. J. Trop. M. and Hyg., Lond., v. 10, Mar., pp. 82-83.

Dock, George, 1898.—Intestinal parasites. Am. System Pract. Med. (Loomis & Thompson), N. Y. and Phila., v. 3, pp. 315-349.

Dunlap, Fay M., 1898.—Tapeworm in abdominal cavity. Med. News, N. Y., v. 72, pp. 118-120.

Edsall, David L., 1904.—Two cases of *Bothriocephalus latus* infection, one of them showing profound anemia. Am. Med., Phila., v. 8 (26), Dec. 24, p. 1087-1091.

Ewing, James, 1903.—[*Bothriocephalus* in a Swede. Abstract.] Proc. N. York Path. Soc., n. s., v. 2, Jan., p. 151.

Fetterdolf, D. W., 1907.—The existence of an antienzyme in tapeworm. Univ. Penn. M. Bull., Phila., v. 20, pp. 94-96.

Gerhard, Samuel P., 1903.—Treatment of tapeworm. Med. News, N. Y., v. 83, p. 920.

Gottman, Abraham, 1898.—Chorea due to tapeworm. Med. Rev. of Reviews, N. Y., v. 4, pp. 539-540.

Gumbiner, A. A., 1906.—Tapeworm simulating appendicitis recurrens. N. York M. J. (etc.), v. 83, pp. 349.

Haglestam, Jarl, 1896.—Anemia caused by tapeworm (*Bothriocephalus latus*) observed in the United States. N. York M. J., v. 64, Aug. 29, pp. 289-291.

Hallock, H. M., 1904.—*Tenia nana:* Report of two cases. J. Am. M. Ass., Chicago, v. 42, p. 891.

Hardin, C. B., 1907.—Some remarks on the *Tænia* group. Kansas City M. Index-Lancet, v. 28, pp. 11-17.

Hensley, J. L., 1899—*Tænia solium.* Tr. Ohio Eclectic M. Ass., Columbus, v. 35, pp. 147-150.

Huber, Francis, 1908.—*Bothriocephalus latus.* N. York M. J. (etc.), v. 87, p. 1190.

Janeway, Theodore C., 1903.—[Case of *Bothriocephalus latus* in a woman from Finland. Abstract.] Proc. N. York Path. Soc., n. s., v. 2, Jan., p. 150.

Kime, J. W., 1899.—Treatment of tapeworm by use of morphine injected into the protruding part of the parasite. Gaillard's M. J., N. Y., v. 71, p. 663.

Lambert, Sámuel W., 1906.—Infection by *Tænia nana,* with specimens. Med. Rec., N. Y., v. 70, Dec. 8, p. 928.

Lapowski, B., 1899.—A case of spermatorrhea probably due to *Tænia solium.* J. Cutan. and Genito-Urin. Dis., N. Y., v. 17, pp. 577-578.

Leech, W. Stuart, 1905.—*Tænia.* Wisconsin M. Recorder, Janesville, v. 8, pp. 319-320.

Lippe, M. J., 190.—*Tænia* in a child. St. Louis Courier of Med., v. 30, June, pp. 336-337.

Lucas, C. C., 1909.—*Tænia.* Kentucky M. J., Bowling Green, v. 7, p. 218.

Lynah, H. L., 1907.—A case of anemia due to *Bothriocephalus latus.* Proc. N. York Path. Soc., v. 7, pp. 91-92.

McFarland, Joseph, 1902.—A case of *Bothriocephalus latus*. Medicine, Detroit, v. 8, pp. 469-470.

Moore, John T., 1903.—The occurrence of *Tænia nana* in Texas (the first or at least the second reported case in North America). Tr. Texas M. Ass., Austin, pp. 577-585.

Nickerson, W. S., 1906.—The broad tapeworm in Minnesota, with the report of a case of infection acquired in the State. J. Am. M. Ass., Chicago, v. 46, Mar. 10, pp. 711-713.

Packard, F. A., 1891.—Specimen of *Bothriocephalus latus*. Tr. Phila. Path. Soc., Phila., v. 14, pp. 62-63.

Ransom, Brayton Howard, 1904.—An account of the tapeworms of the genus *Hymenolepis* parasitic in man, including reports of several new cases of the dwarf tapeworm (*H. nana*) in the United States. Bull. No. 18, Hyg. Lab., P. H. and Mar. Hosp. Serv., Wash., 138 pp.

1908.—Occurrence of the *Cysticercus* of *Tænia solium* in sheep. Science, N. Y., n. s. (703), v. 27, June 19, pp. 950-951.

Riesman, David, 1902.—A specimen of *Bothriocephalus latus*. Medicine, Detroit, v. 8, Feb., pp. 112-116.

Rosenberger, R. C., 1903.—A peculiar teratologic form of *Tænia saginata*. Am. Med., Phila., v. 6, p. 93.

Schaumann, Ossian, 1900.—Die perniciöse Anämie im Lichte der modernen Gifthypothese. Samml. f. klin. Vortr., Leipz., n. F., No. 287, Inn. Med., No. 84, Dec., pp. 231-282.

Shivers, M. O., 1900.—A tapeworm of unusual length. Memphis M. Month., v. 20, Aug., pp. 419-420.

Spier, Seymour L., 1905.—*Tænia* and its treatment. Yale M. J., N. Haven, v. 11, pp. 338-343.

Spooner, E. A., 1873.—Specimens of *Tænia nana*. Tr. Coll. Phys., Phila., n. s., v. 4, p. 416.

Stengel, 1902.—Two cases of *Dibothriocephalus latus*. [Abstract.] Proc. Path. Soc. of Phila., n. s., v. 5, Jan., p. 87.

Stiles, Charles Wardell, 1895.—Rarity of *Tænia solium* in North America. Vet. Mag., N. Y., v. 2, pp. 281-286.

1903.—The dwarf tapeworm (*Hymenolepis nana*), a newly recognized and probably rather common American parasite. N. York M. J. (etc.), v. 78, pp. 877-881.

1905.—Tæniasis—Cestode infection. Modern Med., Phila. and N. Y. (Osler & McCrae), v. 1, pp. 556-581.

Stucky, Thomas Hunt, 1905.—Tapeworm. Louisville Month. J. M. and S., v. 12, Dec., pp. 210-211.

Thompson, W. Gilman, 1905.—A case of *Dibothriocephalus latus* infection, causing pernicious anemia; with complete recovery. Med. News, N. Y., v. 86, Apr. 8, pp. 635-638.

Van Nuys, Franklin B. (M. D., Lakeview), 1907.—*Bothriocephalus latus*. Am. M. Compend., Toledo, v. 23, Aug., pp. 254-255.

Walker, John T., 1879.—*Bothriocephalus latus*. Phila. M. Times, v. 9, Apr. 12, p. 326.

Willson, Robert N., 1902.—*Bothriocephalus latus;* report of a case of double infection, with a discussion of the causation of primary and secondary pernicious anemia. Am. J. M. Sc., Phila. and N. Y., n. s., v. 124, Aug., pp. 262-286.

Wilson, R. L., 1908.—A case of multiple tapeworm infection. N. York M. J. (etc)., v. 87, pp. 743-744.

---

When a patient with inflamed varicose veins develops suddenly dyspnea and cyanosis, don't sit her up to examine her—the probability of pulmonary embolism is too great.—*American Journal of Surgery.*

